# Preoperative Proteinuria Is Associated with Long-Term Progression to Chronic Dialysis and Mortality after Coronary Artery Bypass Grafting Surgery

**DOI:** 10.1371/journal.pone.0027687

**Published:** 2012-01-20

**Authors:** Vin-Cent Wu, Tao-Min Huang, Pei-Chen Wu, Wei-Jie Wang, Chia-Ter Chao, Shao-Yu Yang, Chih-Chung Shiao, Fu-Chang Hu, Chun-Fu Lai, Yu-Feng Lin, Yin-Yi Han, Yih-Sharng Chen, Ron-Bin Hsu, Guang-Huar Young, Shoei-Shen Wang, Pi-Ru Tsai, Yung-Ming Chen, Ting-Ting Chao, Wen-Je Ko, Kwan-Dun Wu, the NSARF Group

**Affiliations:** 1 Division of Nephrology, Department of Internal Medicine, National Taiwan University Hospital, Taipei, Taiwan; 2 Division of Nephrology, Department of Internal Medicine, Yun-Lin Branch, National Taiwan University Hospital, Douliou, Taiwan; 3 Division of Nephrology, Department of Internal Medicine, Da Chien General Hospital, Miaoli, Taiwan; 4 Department of Internal Medicine, Taoyuan General Hospital, Department of Health, Executive Yuan, Taoyuan, Taiwan; 5 Department of Surgery, National Taiwan University Hospital, Taipei, Taiwan; 6 Division of Nephrology, Department of Internal Medicine, Saint Mary's Hospital, and Saint Mary's Medicine, Nursing, and Management College, Luodong, Yilan; 7 International Harvard Statistical Consulting Company, Taipei, Taiwan; 8 Department of Raumatology, National Taiwan University Hospital, Taipei, Taiwan; 9 Medical Research Center, Cardinal Tien Hospital, Fu Jen Catholic University College of Medicine, Taipei, Taiwan; University of Chicago, United States of America

## Abstract

**Aims:**

Preoperative proteinuria is associated with post-operative acute kidney injury (AKI), but whether it is also associated with increased long- term mortality and end -stage renal disease (ESRD) is unknown.

**Methods and Results:**

We studied 925 consecutive patients undergoing CABG. Demographic and clinical data were collected prospectively, and patients were followed for a median of 4.71 years after surgery. Proteinuria, according to dipstick tests, was defined as mild (trace to 1+) or heavy (2+ to 4+) according to the results of the dipstick test. A total of 276 (29.8%) patients had mild proteinuria before surgery and 119 (12.9%) patients had heavy proteinuria. During the follow-up, the Cox proportional hazards model demonstrated that heavy proteinuria (hazard ratio [HR], 27.17) was an independent predictor of long-term ESRD. There was a progressive increased risk for mild proteinuria ([HR], 1.88) and heavy proteinuria ([HR], 2.28) to predict all–cause mortality compared to no proteinuria. Mild ([HR], 2.57) and heavy proteinuria ([HR], 2.70) exhibited a stepwise increased ratio compared to patients without proteinuria for long–term composite catastrophic outcomes (mortality and ESRD), which were independent of the baseline GFR and postoperative acute kidney injury (AKI).

**Conclusion:**

Our study demonstrated that proteinuria is a powerful independent risk factor of long-term all-cause mortality and ESRD after CABG in addition to preoperative GFR and postoperative AKI. Our study demonstrated that proteinuria should be integrated into clinical risk prediction models for long-term outcomes after CABG. These results provide a high priority for future renal protective strategies and methods for post-operative CABG patients.

## Introduction

Although coronary artery bypass grafting (CABG) surgery can result in improved quality and prolongation of life in selected patients, several factors have been identified as independent predictors of poor outcomes [1]. The pre-operative estimated glomerular filtration rate (eGFR) is one of the most powerful predictors of CABG outcome [2], [3]. In addition, even a mild elevation of serum creatinine after surgery carries a significant risk of adverse outcomes [4].

Based on experience with chronic kidney disease (CKD), proteinuria (detected either by dipstick tests or the albumin-creatinine ratio [ACR]) has been shown to be strongly associated with adverse outcomes, including incident acute kidney injury (AKI), renal disease progression, cardiovascular events, and long-term mortality in the general population [5]–[8]. Recent reports from large epidemiologic studies have shown that patients with proteinuria have a higher risk of adverse outcomes than those without proteinuria at the same stage of CKD [9], [10]. It has been suggested that GFR and proteinuria should be used together to identify patients at risk [5], [9]–[11]. Screening for proteinuria is a better strategy than relying on a low eGFR to identify individuals who are at risk for accelerated GFR loss in population screening [12]. Furthermore, the episode of AKI could provide further long-term prognostic information in addition to eGFR and proteinuria [11]. We previously reported that pre-operative proteinuria, independent of pre-operative eGFR and other co-morbidities, could predict AKI in patients undergoing CABG [13]. No study has shown pre-operative proteinuria to be an independent risk factor of long-term mortality and dialysis dependence in post-CABG patients with a high mortality rate.

The objective of this study was to estimate the post-discharge, long-term mortality, and ESRD risk associated with pre-operative proteinuria, while adjusting for pre-operative eGFR with post-operative AKI and other co-morbidities in post-CABG patients.

## Results

Among the 1136 adult patients who underwent CABG during the period from January 2003 to December 2006, 50 patients had undergone dialysis before surgery, 30 patients were CKD stage 5 before surgery, and 76 patients did not have urinalysis measurements before surgery. During the hospital admission, 55 patients expired. Therefore, only 925 patients were included in the final analysis (Figure S1).

### Patients with impaired renal function (low eGFR or proteinuria; Table 1, 2)

**Table 1:** Patients' demographics, classified by CKD stages or proteinuria.

		*CKD Stage*			*Proteinuria on dipstick*		
	All (n = 925)	*Preserved eGFR*	*Stage 3*	*Stage 4*	*Normal*	*Mild*	*Heavy*
		*(n = 554)*	*(n = 319)*	*(n = 52)*	*(n = 530)*	*(n = 276)*	*(n = 119)*
**Patient characteristics**							
Gender (male)	75.9%	82.3%	69.6%***	46.2%***	79.2%	75.7%	61.3%***
Age (years)	65.9±10.9	63.5±11.3	69.6±8.6***	69.0±10.9***	65.1±11.4	67.3±9.9*	66.2±10.1
Body mass index (kg/m^2^)	25.0±3.7	25.1±3.3	25.1±4.2	24.3±3.8	25.1±3.4	25.4±4.2	24.2±3.5
Charlson score	1.8±1.8	1.5±1.5	2.2±2.0***	3.3±1.6***	1.5±1.5***	2.0±1.9***	3.0±2.2***
LVEF<60%	46.9%	42.1%	49.8%*	80.8%***	40.8%	51.1%**	64.7%***
Hypertension	69.2%	65.9%	74.6%**	71.2%	68.1%	69.9%	72.3%
DM	43.4%	38.4%	48.6%**	63.5%***	34.9%	44.6%**	78.2%***
PAD	9.0%	6.9%	9.4%	28.8%***	6.4%	8.3%	21.8%***
CVA	9.9%	8.8%	11.0%	15.4%*	7.9%	11.6%	15.1%*
CHF	15.9%	11.2%	19.4%***	44.2%***	10.4%	19.2%***	32.8%***
COPD	8.1%	7.6%	9.4%	5.8%	7.2%	11.2%	5.0%
Recent MI	26.3%	24.0%	28.5%	36.5%	23.4%	32.2%**	25.2%
Af	6.6%	5.4%	9.4%*	1.9%	5.1%	8.7%	8.4%
**Peri-operative condition**							
Vasopressor dependence	3.6%	2.7%	4.2%*	9.7%**	2.2%	5.7%^**^	5.1%
Tracheostomy	2.2%	1.1%	3.4%*	5.8%*	0.9%	2.5%	6.7%***
Non-elective surgery	10.5%	9.4%	11.7%	13.5%	6.8%	15.4%***	15.1%**
Cardiopulmonary bypass	13.6%	12.5%	14.1%	23.1%*	11.3%	15.9%	18.5%*
AKI	15.1%	9.9%	20.4%***	38.5%***	9.6%	19.6%***	29.4%***
RRT	3.6%	2.0%	3.5%	22.4%***	1.5%	2.2%	16.4%***

**Table 2:** Operative characteristics and renal function, classified by CKD stages or proteinuria.

		*CKD Stage*			*Proteinuria on dipstick*		
	All (n = 925)	*Preserved eGFR*	*Stage 3*	*Stage 4*	*Normal*	*Mild*	*Heavy*
		*(n = 554)*	*(n = 319)*	*(n = 52)*	*(n = 530)*	*(n = 276)*	*(n = 119)*
**CABG parameters**							
Aortic cross clamp time(min),(n)	103± 41 (31)	82± 36 (15)	101± 58 (13)	102± 23 (3)	105± 65 (12)	108±36 (14)	97± 25 (5)
Cardiopulmonary bypass(min),(n)	125± 53 (130)	127±51 (75)	123± 71 (42)	127± 41 (13)	125± 60 (60)	128± 63 (48)	120± 32 (22)
Triple vessel disease	93.4%	81.2%	86.2%	88.5%	81.7%	87.0%	92.4%
Left main diseases	37.8%	38.8%	35.4%	42.3%	37.5%	42.0%	29.4%
IABP	9.1%	7.8%	11.0%	11.5%	7.7%	10.9%	10.9%
Preoperative IABP	7.4%	6.1%	9.4%	7.7%	5.5%	9.4%	10.9%
ECMO	1.2%	0.9%	1.3%	3.8%	0.6%	2.2%	1.7%
Mitral insufficiency							
Mild	18.9%	19.9%	17.2%	19.2%	19.1%	20.7%	14.3%
Moderate/severe	8.6%	7.6%	8.2%	23.1%***	5.8%	9.4%	19.3%
**Pre-operative laboratory data**							
Creatinine (mg/dL)	1.3±0.5	1.0±0.2	1.5±0.3***	2.6±0.55***	1.12±0.4	1.3±0.4**	1.7±0.7***
eGFR(ml/min/1.73 m^2^)	64.4±21.1	77.6±14.6	48.12±8.4***	23.22±4.02***	69.±18.7	62.3±21.2***	48.10±22.0***
Hemoglobin (g/dL)	12.9±1.9	13.4±1.6	12.5±2.06***	10.9±1.8***	13.2±1.8	12.8±1.9*	11.9±1.9***

Comparison with patients with preserved estimated GFR (≥60 ml/min/1.73 m^2^) or no proteinuria: *p<0.05; **p<0.01; and ***p<0.001.

Abbreviations; CABG: coronary artery bypass grafting surgery; CKD: chronic kidney disease; eGFR: estimated glomerular filtrating rate; ESRD: end stage renal disease.

Three hundred nineteen patients (34.5%) were classified as CKD stage 3, while 52 (5.6%) patients were classified as stage 4 pre-operatively. The patients with higher CKD stages (stages 3 and 4) were older and had higher Charlson scores, lower pre-operative Hb levels, required more tracheostomies, had more post-cardiac surgery AKI than patients with preserved kidney function. Patients with stage 4 CKD were more likely to have PAD, undergo CPB, and required post-operative RRT than patients with preserved kidney function.

A total of 276 (29.8%) patients had mild proteinuria before surgery and 119 (12.9%) patients had heavy proteinuria. Those with proteinuria were more likely to have DM, impaired left ventricular contractility, CHF, and post-operative AKI. These patients also had significantly lower eGFRs, higher Charlson scores, and lower pre-operative hemoglobin (Hb) levels than non-proteinuric patients. Patients with heavy proteinuria were more likely to have PAD, CVA, tracheostomies, receive CPB, and require post-operative RRT.

In patients with preserved eGFR, 147 (15.9%) patients had mild proteinuria and 34 (3.7%) patients had heavy proteinuria. In stage 3 CKD patients, 11 (15.6%) had mild proteinuria and 65 (18.1%) had heavy proteinuria, and in stage 4 patients, 12 (4.3%) had mild proteinuria and 27 (22.7%) had heavy proteinuria (Table S1).

Long-term adverse outcomes were stratified by CKD stage and the severity of proteinuria (Table 3). Patients with preserved eGFR and without proteinuria had the lowest rates of post-operative RRT (0.3%), mortality (5.6%), and composite outcomes (5.9%). The severity of proteinuria showed a dose response-type increased risk for adverse long-term outcomes in patients with preserved eGFR, and CKD stage 3, but not in patients with CKD stage 4.

**Table 3:** Adverse outcomes in 925 CABG patients with various CKD stages and degrees of proteinuria.

Outcomes	ESRD (n = 41)			*p* ^b^	Mortality (n = 138)			*p* ^b^	Composite outcome (n = 164)			*p* ^b^
Proteinuria												
CKD Stages	Normal	Mild	Heavy		Normal	Mild	Heavy		Normal	Mild	Heavy	
**Preserved eGFR**	0.3%	1.4%	11.8%	<0.001	5.6%	15.6%	14.7%	0.001	5.9%	17.0%	26.5%	<0.001
**(n = 554)**												
**Stage 3**	1.4%	2.6%	17.2%	<0.001	15.3%	24.8%	34.5%	0.009	16.7%	26.5%	43.1%	<0.001
**(n = 319)**												
**Stage 4**	23.1%	58.3%	38.5%	0.195	30.8%	41.7%	48.1%	0.580	46.2%	75.0%	66.7%	0.288
**(n = 51)**												
***p^a^***	<0.001	<0.001	0.028		<0.001	0.035	0.018		<0.001	<0.001	0.007	

Abbreviations; CABG: coronary artery bypass grafting surgery; CKD: chronic kidney disease; eGFR: estimated glomerular filtrating rate; ESRD: end stage renal disease.

*p*: The tests for linear trend across CKD categories (*p* ^a^) and across proteinuria categories (*p* ^b^). Composite outcome: composite outcome of end stage renal disease and mortality. To be noted, 15 patients of them received chronic dialysis before mortality.

### ESRD after CABG (Table 4, Fig. 1)

**Figure 1:**
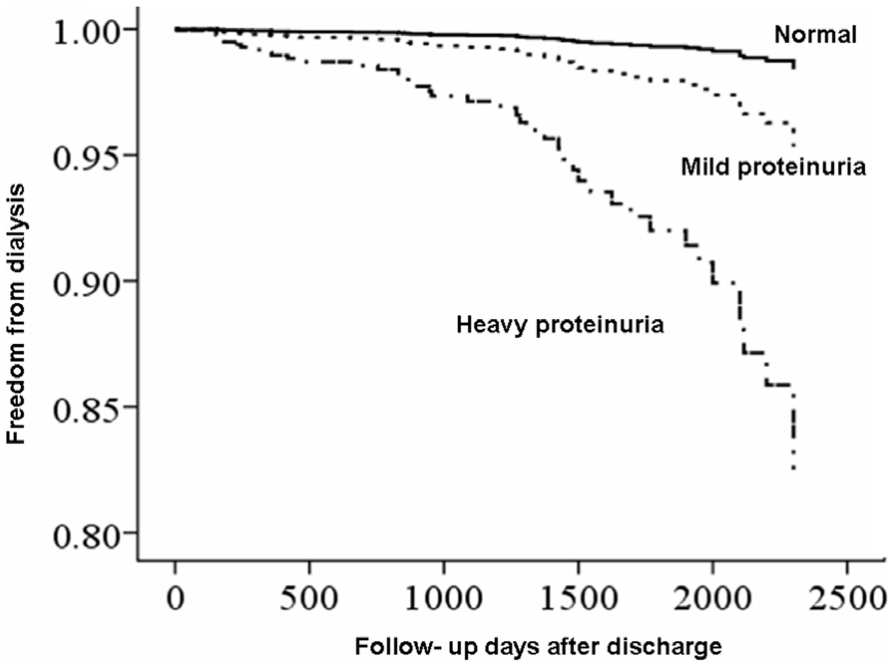
Proportion of freedom from long- term dialysis dependence, stratified by different severities of proteinuria defined by preoperative dipstick. (Mild proteinuria, p = 0.166; Heavy proteinuria, p = 0.001; No proteinuria was the reference calculated by multivariable Cox proportional hazard analyses).

**Table 4:** Factors associated with long- term adverse outcomes (N = 925).

Hazard Ratio(95% CI)	ESRD		all cause mortality		composite outcome	
Covariate	Unadjusted	adjusted	Unadjusted	adjusted	Unadjusted	adjusted
***Proteinuria***						
**No proteinuria**	1	—	—	—	—	—
	(reference)					
**Mild proteinuria**	3.93	2.83	2.29	1.88	2.42	2.70
	(1.48–10.40)**	(1.01–7.99)*	(1.55–3.39) ***	(1.27–2.80)*	(1.67–3.49) ***	(1.69–4.33)***
**Heavy proteinuria**	10.10	27.17	2.65	2.28	3.36	2.57
	(3.98–25.73)***	(8.77–84.15)**	(1.68–4.18)***	(1.42–3.66)**	(2.23–5.06) ***	(1.68–3.91)***
***CKD Stage***						
**Preserved CKD stage**	1	—	—	—	—	—
	(reference)					
**Stage 3**	3.29	10.71	2.35	1.53	2.33	2.24
	(1.32–8.15)*	(0.93–123.2)	(1.62–3.41) ***	(1.20–2.28)*	(1.64–3.30) ***	(1.40–3.57)***
**Stage 4**	35.52	91.21	4.85	1.88	7.35	3.52
	(14.25–88.52)***	(31.05–267.94)***	(2.83–8.33) ***	(1.03–3.43)*	(4.65–11.63)***	(2.09–5.94)***

Abbreviations: CI: confidence interval; CKD: chronic kidney disease.

The long- term adverse outcome uses the Cox's proportional hazard model adjusted for age, genders, admission conditions including CKD stage, postoperative acute kidney injury, renal replacement therapy, co-morbidities (hypertension, liver cirrhosis, congestive heart failure, diabetic mellitus, chronic obstructive pulmonary disease , coronary artery disease, hepatitis, cancer, atrial fibrillation), Carlson score, intervention (extracorporeal membrane oxygenation, Ventilator, intra-aortic balloon pumping, use intra-cerebral pressure monitor, Mitral insufficiency, temporary cardiac pacemaker, Swan- Ganz catheter, Sengstaken-Blakemore tube).

*p<0.05;

**p<0.01; and

***p<0.001.

During follow-up, the total incidence of ESRD was 0.2, 1.1, and 5.5 per 100 person–years among surviving hospital patients with pre-operative normal, mild, and heavy proteinuria, respectively. We included the variables listed in Table 1, 2 into regression analysis to identify significant factors associated with post-operative ESRD. CKD stages were representative of pre-operative renal function, as stated in the Methods. The Cox proportional hazards model demonstrated heavy proteinuria (HR, 27.17) and CKD stage 4 (HR, 91.21) as independent predictors of long-term ESRD (Table S2). The interaction term between CKD and proteinuria (p<0.001) was inverse related to long-term ESRD. The magnitude of the increases associated with ESRD grew progressively with heavier baseline proteinuria. (HR = 5.282, p<0.001 for trend)

### All-cause mortality after CABG (Table 4, Fig. 2)

**Figure 2:**
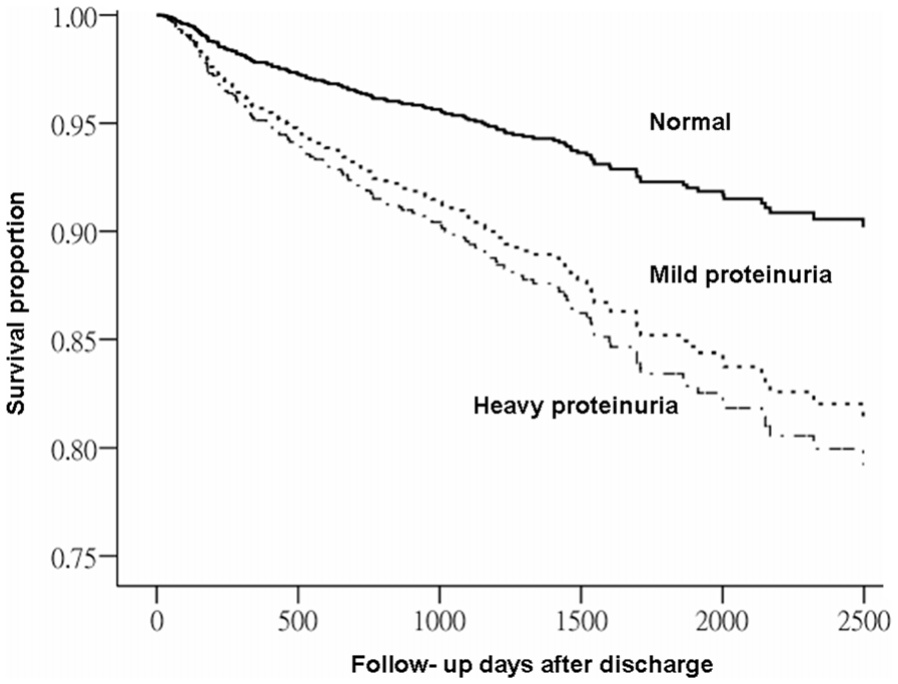
Adjusted risk for long- term all- cause mortality after hospital discharge stratified by different severities of proteinuria defined by preoperative dipstick. (Mild porteinuria, p = 0.005; Heavy proteinuria, p = 0.008; no proteinuria was the reference calculated by multivariable Cox proportional hazard analyses).

The incidence for all-cause mortality was 1.9, 5.1, and 8.0 per 100 person–years among patients with normal, mild, and strong proteinuria, respectively.

We also put all the variables listed in Table 1, 2 into regression analysis for identifying important risk factors of post-operative mortality. In the final model, there was a progressive increased risk of mild proteinuria (HR, 1.88) and heavy proteinuria (HR, 2.28) to predict all–cause mortality in addition to CKD stages and postoperative AKI. (Table S3). The other significant factors of long-term mortality were older age, low LVEF, a history of CAD, absence of HTN, and post-operative RRT requirements.

The interaction term between CKD and proteinuria was not significant (p>0.05). (Table S3). The test for linear trend across proteinuria categories was significant. (HR = 1.542 , p<0.001).

### Post-operative long-term composite outcomes

**Figure 3:**
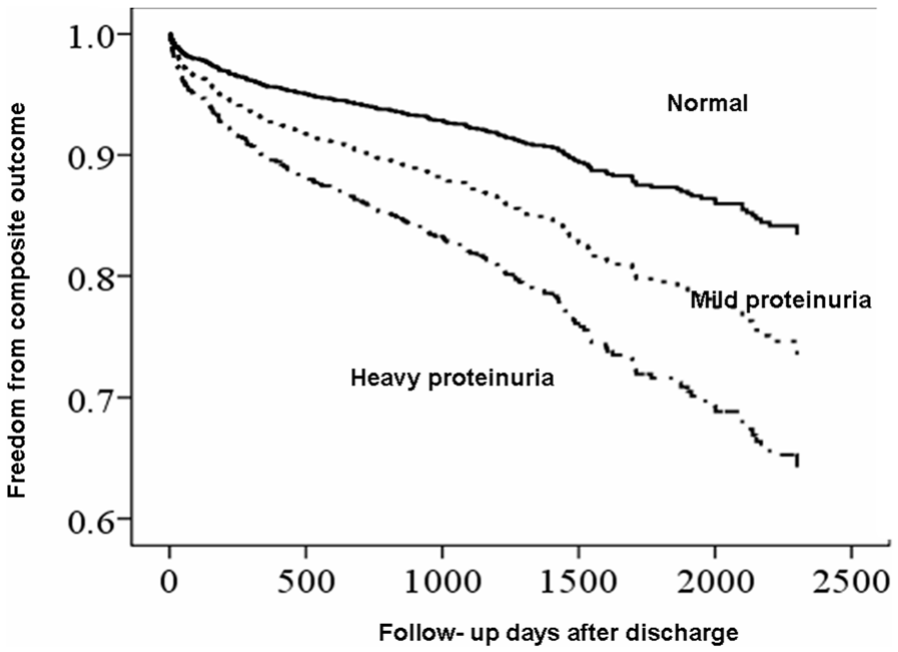
Proportion of freedom from long- term composite outcome after hospital discharge, composite outcome of ESRD and mortality, stratified by different severities of proteinuria defined by preoperative dipstick. (Mild proteinuria, p<0.001; Heavy proteinuria, p<0.001; No proteinuria was the reference calculated by multivariable Cox proportional hazard analyses).

HRs for long–term composite outcomes associated with the CKD and proteinuria categories are shown in Fig. 3. Patients with mild (HR, 2.57) and heavy proteinuria (HR, 2.70) and patients with CKD stage 3 (HR, 2.24) and stage 4 (HR, 3.52) had a stepwise increased ratio compared to patients without proteinuria or patients with preserved eGFR stages (Table S4).

**Figure 4:**
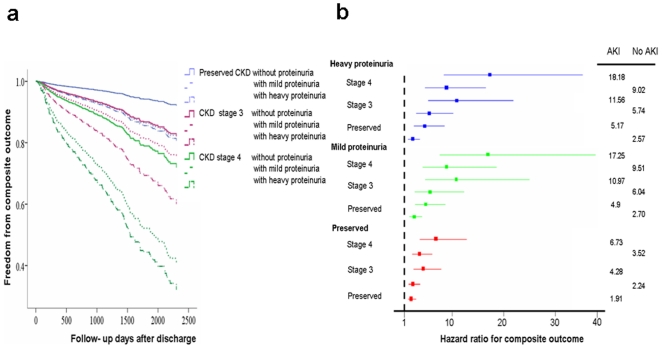
The composite outcome after hospital discharge (long- term end-stage renal disease or mortality) for urinary proteinuric categories across chronic kidney disease (CKD) categories using Cox proportional hazards regression (a) plot of freedom from composite outcome, ^*^ *p*<0.05; ^**^ *p*<0.01; and ^***^ *p*<0.001 compared to patients with preserved eGFR and normal proteinuria. (b) Hazard ratio (HRs) stratified by proteinuria, baseline kidney function, and postoperative acute kidney injury (AKI) adjusted for factors listed in Table 1. The horizontal bars represent 95% CIs for HRs of participants who had proteinuria for various values of CKD stages and AKI.

HRs were obtained using Cox proportional hazards regression for categorical analysis and adjusted for factors listed in Table 1, 2 with CKD stages as representative of pre-operative kidney function (Fig. 4). Figure 4a showed survival curves for proteinuria categories across eGFR categories. The HRs for long-term composite outcome increased with the severity of proteinuria (Figure S2), and also increased with CKD stages. Both CKD stages and postoperative AKI modified the frequency and consequences of proteinuria status. (Figure 4b) The HR for composite outcome was the highest with heavy proteinuria strata within stage 4 CKD (HR, 11.58; 95% CI, 5.20–26.70; p<0.001). The risk for composite outcome was magnified further when mild proteinuria was present in those with baseline stage 4 CKD (HR, 9.9; 95% CI, 4.83–20.30; p<0.001) compared to patients with a preserved CKD stage without proteinuria. The magnitude of the increases associated with composite outcome grew progressively with heavier baseline proteinuria. (HR = 1.649 , p<0.001 for trend).

Several sensitivity analyses were undertaken. We compared the characteristics of the 76 patients without pre-operative urinalysis with the patients included in the data analysis. Similar results were noted with respect to age (p = 0.870), gender (p = 0.799), CKD stages (p = 0.786), DM (p = 0.250), long-term ESRD (p = 0.999), long-term mortality (p = 0.327), and long-tem composite outcome (p = 0.647). DM had an additive interaction with CKD stages in predicting long-term mortality. Because of this, we repeated our logistic regression model stratified by diabetes status. Heavy proteinuria was associated with an even greater risk of mortality among patients with diabetes (HR, 3.34) than among those without (HR, 1.88) diabetes.

## Discussion

We have found that proteinuria is potentially a risk factor of postoperative adverse outcome; however, this simple test is usually neglected in current practice. These findings should make clinicians more concerned about the presence of low quantities of protein in the urine. Our study is the first report to show a marked impact of proteinuria at the time of CABG on long-term mortality and ESRD prediction in post-CABG patients. The National Kidney Foundation staging system for CKD incorporates proteinuria only in the preserved eGFR stages (CKD stages 1 and 2). An association between proteinuria and long- term mortality or ESRD after cardiac surgery has not yet been validated. We showed that proteinuria is associated with a higher risk of long-term ESRD and mortality; even mild proteinuria could predict long- term adverse outcomes. This association was essentially unaltered despite extensive statistical adjustment for traditional CABG risk factors, including chronic kidney disease, AKI, or diabetes mellitus. This adds weight to the growing body of evidence that proteinuria should be considered a high risk condition and that this risk is in addition to any risk attributable to reduced eGFR [5].

The results clearly demonstrated that pre-operative proteinuria is an important, yet neglected predictor for long-term composite outcomes after cardiac surgery. However, the current widely used prognostic scoring systems do not use proteinuria [14]. Preventive interventions have been most studied in patients at high risk because high-risk patients benefit more from such interventions than low-risk patients [15]. Thus, it is very important to develop an inclusive and accurate staging system, especially for patients who are undergoing a procedure associated with significant risks, such as cardiac surgery. Also, this is the first report to systemically investigate the relationship between proteinuria and post-operative long –term renal outcomes and demonstrate the strong association with adverse renal outcomes.

### Risk for ESRD according to severity of proteinuria

We have previously reported heavy proteinuria, regardless of baseline eGFR or other co-morbidities, to be independently associated with severe AKI requiring RRT after cardiac surgery [13]. Proteinuria reflects a size-selective dysfunction of the glomerular barrier usually associated with a decline in the GFR that may result in ESRD [16]. There is some evidence that overt albuminuria might be associated with tubulointerstitial damage. Patients with documented proteinuria have less physiologic adaptability and are less able to tolerate kidney hemodynamic changes and other nephrotoxic insults [17].

In a previous population-based cohort study, macroalbuminuria is a better risk marker than low eGFR to identify population screening of individuals who are at risk for accelerated GFR loss [12]. In fact, our CABG patients with preserved kidney function and positive dipstick proteinuria have a greater risk for having a composite outcome than patients with stage 3 CKD and without proteinuria detected by dipstick.

### Risk for mortality according to severity of proteinuria

We found the association between eGFR and mortality differed by severity of proteinuria. Decreased eGFR and increased proteinuria independently contributed to the cumulative probability of all-cause mortality and the risk persisted after full multivariable adjustments, including post-operative AKI. Previous studies have demonstrated that AKI is associated with increased long-term mortality after cardiothoracic surgery, even with a small change in serum creatinine [18]. Further, pre-operative proteinuria and CKD stages, independent of post-operative AKI, are associated with an increased risk of long–term mortality.

When the analysis focused on post-operative composite outcome, patients with impaired eGFR and proteinuria were found to have a greater risk than those without proteinuria. Even mild proteinuria (trace to 1+) increased the risk of a patient with stage 3 CKD to the same risk as stage 4 CKD (Figs. 4). Most strikingly, patients with preserved GFR (≥60 mL/min/1.73 m^2^) had a risk comparable to stage 3 CKD, if mild proteinuria (trace to 1+) was present (Fig. 4). These categories of patients have previously been neglected, even though they made up 32.7% (181 of 554 patients) of our cohort. This means that one-third of patients undergoing CABG surgery, who are at an increased risk of long-term mortality and ESRD, are not identified by the current risk scoring systems based only on sCr or GFR measurements. On the other hand, the absence of an interaction effect of CKD and proteinuria in the prediction of long-term mortality was notable because a very small amount of protein in urine was associated with a significant risk of mortality in the CKD patients [19]. These findings should motivate clinicians to become more concerned about the presence of low quantities of protein in the urine.

A previous small study about CABG showed proteinuria is factor to affect long-term cardiovascular death [20]. However, CKD was not a risk factor in their study. Bouts of evidence show the pre-operative estimated glomerular filtration rate (eGFR) is one of the most powerful predictors of CABG outcome [2], [3]. In addition, we report that proteinuria is a powerful independent risk factor of long-term all-cause mortality and ESRD after CABG in addition to preoperative GFR.

The association between higher CKD stages and higher long-term cumulative composite outcomes increased significantly with an increased severity of proteinuria. In linear trend analysis, the severity of proteinuria showed a dose response-type increasing risk for long-term outcomes in patients with preserved eGFR, and CKD stage 3, but not for stage 4. ( please see table 3). However, in multivariable model the HRs for long-term composite outcome increased with the severity of proteinuria (Figure S2), and also increased with CKD stages. This association was observed in participants with and without a history of DM in sensitivity analysis and this result could enhance the joint effects of proteinuria and CKD stages.

Pre-operative proteinuria is not only a marker of chronic renal insults, but may also serve as a surrogate of organ damage. Patients with more severe proteinuria had higher Charlson scores. In our analysis, patients with more severe proteinuria had poor cardiac contractility, DM, PAOD, and CHF, which implies that these patients actually have renal damage and proteinuria secondary to other extra-renal insults. Proteinuria may not just be a marker for adverse outcomes. *In vitro* studies have demonstrated that exposure of renal proximal tubule cells to albumin induced an inflammatory cytokine cascade [21], [22]. These events ultimately lead to tubulointerstitial inflammation and fibrosis in long-standing proteinuric nephropathy. The strong predictive effects of proteinuria within 2 days before surgery and the simplicity of its measurements suggest that periodic measurement of proteinuria along with other major CVD risk factors should be considered in long-term follow-up of post CABG patients. Whether or not medications that decrease proteinuria might also be associated with a decrease in mortality in persons undergoing CABG is an important question to be addressed in future studies.

In a large community –based cohort of adults, the risk of long –term mortality composite renal outcome increased substantially with the presence and severity of proteinuria, in addition to reduced eGFR [11]. Similarly, outcomes associated with post CABG AKI have focused on definitions incorporating in proteinuria, magnitude of increase in serum creatinine [13]. Our CABG patients also provide a useful demonstration of the burden of post-operative AKI. (fig. 4b) This disorder was more likely to prognosis to mortality and composite outcomes during long-term follow-up and was substantially more common at low GFR and with heavy proteinuria.

### Study strengths and limitations

The strength of this study was that it assessed the outcomes in a large cohort of patients undergoing CABG in multiple centers. Therefore, the results are likely to be widely applicable. The study population of CABG patients without severe CKD provided a well-defined and homogenous study population in the present study.

There were some limitations to the current study that should be considered. First, detection of proteinuria was performed with dipsticks. In addition, the differences between the dipstick test and ACR in risk assessment of long-term outcomes were not examined in the current study. The epidemiologic studies on this topic either used a dipstick test for proteinuria [11] or quantitatively measured albuminuria. Of note, the urine dipstick examination is inexpensive and readily performed and interpreted. These two tests were well-correlated [23] and the dipstick test is useful for risk stratification despite being a less precise measure of albuminuria in general population cohorts [24], [25]. Second, the absence of data on the specific cause of mortality, such as myocardial infarction and heart failure could be a limitation, thus we used all-cause mortality as the primary end point, which is entirely objective [26]. Finally, we assumed that the baseline covariates persisted throughout the follow-up period. Additional assessments would be desirable, especially to evaluate risks that vary over time. Increased numbers of outcome events, including cause-specific mortality and incident ESRD, will buttress the conclusions presented herein.

The present study demonstrated that proteinuria, detected by urine dipstick test, is a powerful independent risk factor for long-term composite outcomes (all-cause mortality and ESRD) in post–CABG patients. The association between CKD stages and long-term composite outcomes differed significantly across severity of proteinuria. A further strength was that pre-operative proteinuria in addition to pre-operative CKD stages and post-operative AKI is associated with increased long–term mortality risk. A substantial proportion of patients undergoing cardiac surgery have an elevated risk of adverse outcomes that are not apparent from the current risk scoring systems. This is an important insight for physicians who care post-CABG patients, and further studies will be needed to determine the optimal post-discharge follow-up of renal function for patients with pre-operative proteinuria.

## Methods

### Study population

This is a secondary analysis of a prospectively-collected database. Patients undergoing CABG surgery at the National Taiwan University Hospital (NTUH) and its two branches between January 2003 and December 2006 were enrolled [27]. Inclusion criteria were age ≥18 years and first-time cardiac surgery. The exclusion criteria were a history of pre-operative renal replacement therapy (RRT) with any modality and an estimated GFR<15 mL/min. Patients with no urinalysis reports within 48 hours prior to surgery were also excluded. Patient data collected from the NSARF database included basic demographic characteristics, peri-operative laboratory investigations, type and timing of surgery, and post-operative renal outcome [27], [28]. The goals of this study were to evaluate the post-CABG long-term outcomes associated with proteinuria. Patients undergoing pre-operative chronic dialysis, stage 5 CKD, or death during the index hospital admission were excluded. This study was approved by the Institutional Review Board of National Taiwan University Hospital (NTUH) (No. 31MD03). The informed consent was waived by the ethics committee because there was no breach of privacy and it did not interfere with clinical decisions related to patient care.

### Clinical assessment of study patients

Pre-operative variables, such as age, gender, left ventricular ejection fraction (LVEF) measured by ventriculography or angiography, hypertension (HTN; blood pressure ≥140/90 mmHg or using anti-hypertensive medications), diabetes mellitus (DM; using oral hypoglycemic agents or insulin), peripheral artery disease (PAD) determined by clinical diagnosis or imaging results, previous cerebral vascular accident (CVA; ischemic or hemorrhagic), New York Heart Association (NYHA) functional class III or IV congestive heart failure (CHF), chronic obstructive pulmonary disease (COPD) requiring long-term bronchodilators or steroids, recent myocardial infarction (MI), i.e., <30 days before surgery, and pre-operative laboratory data were all collected. Coronary artery disease (CAD) was defined as the diagnosis of ischemic heart disease before admission and positive electrocardiographic findings. Patients with associated diseases were assessed using the Charlson co-morbidity score [29]. Peri-operative vasopressor (adrenaline, dopamine, dobutamine, norepinephrine, or isoproterenol) dependence and use of extracorporeal membrane oxygenation (ECMO) or an intra-aortic balloon pump (IABP) were noted. Patients who underwent emergent or urgent surgical procedures were considered non-elective surgery. Utilization of cardiopulmonary bypass (CPB) and CPB duration during surgery was also recorded.

### Pre-operative GFR and proteinuria

The following 3 parameters were used to represent pre-operative GFR: serum creatinine, eGFR, and CKD stage (preserved eGFR, stage 3 or 4, according to eGFR). The baseline serum creatinine (sCr) was the datum obtained at hospital discharge from the previous admission in those who had more than one admission within 1 year before the index admission [30], or a pre-operative creatinine obtained in pre-operative testing excluding the measurements that were performed in the emergency department [18], [31], [32]. The eGFR in each patient was calculated using the 4-variable MDRD equation [33]. CKD stages were determined using the NKF definition, as follows: 15 mL/min/m^2^≤eGFR<30 mL/min/m^2^ was classified as stage 4; and 30 mL/min/m^2^≤eGFR<60 mL/min/m^2^ was classified as stage 3. Patients with an eGFR≥60 mL/min/m^2^ had preserved GFR.

Proteinuria was measured using a dipstick within 2 days before surgery. To classify the severity of proteinuria, we defined negative as “no proteinuria,” trace to 1+ as “mild proteinuria,” and 2+ to 4+ as “heavy proteinuria.” The test strips were measured by an automatic dipstick autoanalyzer (AUTION MAX, AX-4030; ARKRAY, Inc., Kyoto, Japan) with automatic correction of the specific gravity using a pH test pad in a routine laboratory environment. This classification was adopted in a large epidemiologic study in Alberta, Canada [10]. Although ACR is favored for detection of proteinuria, dipstick examination remains the most convenient and inexpensive choice for screening [10]. If there were more than one measurement in 2 days prior to surgery, we chose the most severe result for analysis.

### AKI and RRT

The definition of AKI was based on the Acute Kidney Injury Network (AKIN) criteria, and has been well-validated in cardiac surgery patients for in-hospital mortality prediction [34]. In the NSARF database, sCr values were recorded daily after surgery. Urine output was recorded every hour in the critical care setting.

### Long-term mortality and dialysis dependence

The long-term outcomes for this analysis were mortality, ESRD, and composite outcome (long–term ESRD or mortality) after discharge. Patient survival after discharge was determined through the databank of the National Health Insurance Research Database (NHIRD) in January 2009 [35]. All-cause mortality was documented by matching unique identity numbers with the NHIRD.

The NHIRD contains health care data from >99% of the entire population of 23.74 million in Taiwan, and it covers all inpatient and outpatient medical benefit claims. We also cross–linked our study population with the nationally comprehensive TAIWAN Society Nephrology registry, which recorded all dialysis patients in the island every 3 months.

### Statistical analysis

Statistical analyses were performed with SPSS for Windows (version 15.0; SPSS, Inc., Chicago, IL, USA). A two-sided *p* value≤0.05 was considered statistically significant. Continuous variables were presented as the mean and standard deviation (mean ± SD). Categorical variables were summarized as the frequency and percentage. A group difference in demographic characteristics was examined between each renal dysfunction group (CKD stages or proteinuria) and the preserved renal function group by two-sample *t*-test or chi-square test as appropriate.

The long-term outcomes were based on Cox's proportional hazard model adjusted for age, gender, admission categories, CKD stage, post-operative AKI, RRT, co-morbidities (HTN, liver cirrhosis, CHF, DM, COPD, CAD, hepatitis, cancer, and atrial fibrillation (Af), Carlson score, intervention (ECMO, mechanical ventilation, IABP, intra-cerebral pressure monitor (ICP), temporary cardiac pacemaker (TCP), a Swan- Ganz catheter, PiCCO, and a Sengstaken-Blakemore tube) and censored on 1 January 2009. Survival curves for all-cause mortality or freedom from dialysis were generated from adjusted Cox models. Survival models were initiated at the time of hospital discharge and followed until death or last follow-up time. There was high collinearity among sCr, estimated GFR (MDRD), and CKD stage, so we adjusted the input variable of the CKD stage to be representative of kidney function to emphasize the effect of pre-operative kidney stage. Yet, there is a significant positive relationship between the level of proteinuria and CKD stage (p<0.001) and the risk of AKI might be different in patients with and without DM [36]. Thus, the interaction effects between proteinuria, CKD stages, postoperative AKI, and diabetics on adverse outcomes were also considered.

For the long-term dialysis, an individual who survived at index discharge was censored at death or at the end of the study period. Hazard ratios (HRs) and 95% confidence intervals (CIs) were derived from the Cox proportional hazard model. The incidence of chronic dialysis and all-cause mortality were stratified for participants with different severities of proteinuria. To help visualize the analysis results, we investigated the relationship between the effects of the level of CKD stages and severity of proteinuria by adding interaction terms and plotted by fitting the basic Cox regression analyses separately for the strata of the level of dipsticks.

Finally, sensitivity analyses were conducted. We compared the basic demography and outcomes of the 76 patients without pre-operative urinalysis with the study patients. A two-sided P value<0.05 was considered to indicate statistical significance.

## Supporting Information

Figure S1 Flow diagram of the study population. ( AKI, acute kidney injury; CABG, coronary artery bypass grafting; CKD, chronic kidney disease; ESRD, end stage renal disease; ICU, intensive care unit).
(TIF)

Figure S2 Hazard ratio (HRs) for the compo site outcome after hospital discharge (long- term end-stage renal disease or mortality) for urinary proteinuric categories across chronic kidney disease (CKD) categories. (adjusted for factors listed in Table 1. ^*^ *p*<0.05; ^**^ *p*<0.01; and ^***^ *p*<0.001 compared to patients with preserved eGFR and normal proteinuria).
(TIF)

Table S1 Percentage of patients in groups stratified by chronic kidney disease (CKD) stage and proteinuria.
(DOCX)

Table S2 Factors associated with long- term end stage renal disease (N = 925).
(DOCX)

Table S3 Factors associated with long- term all- cause mortality (N = 925).
(DOCX)

Table S4 Factors associated with long- term composite outcome (N = 925).
(DOCX)
