# Supplementary figures and images for: Preoperative Proteinuria Is Associated with Long-Term Progression to Chronic Dialysis and Mortality after Coronary Artery Bypass Grafting Surgery
Source: PLoS One. 2012 Jan 20;7(1):e27687. doi: 10.1371/journal.pone.0027687 (PMC3262783; doi:10.1371/journal.pone.0027687)

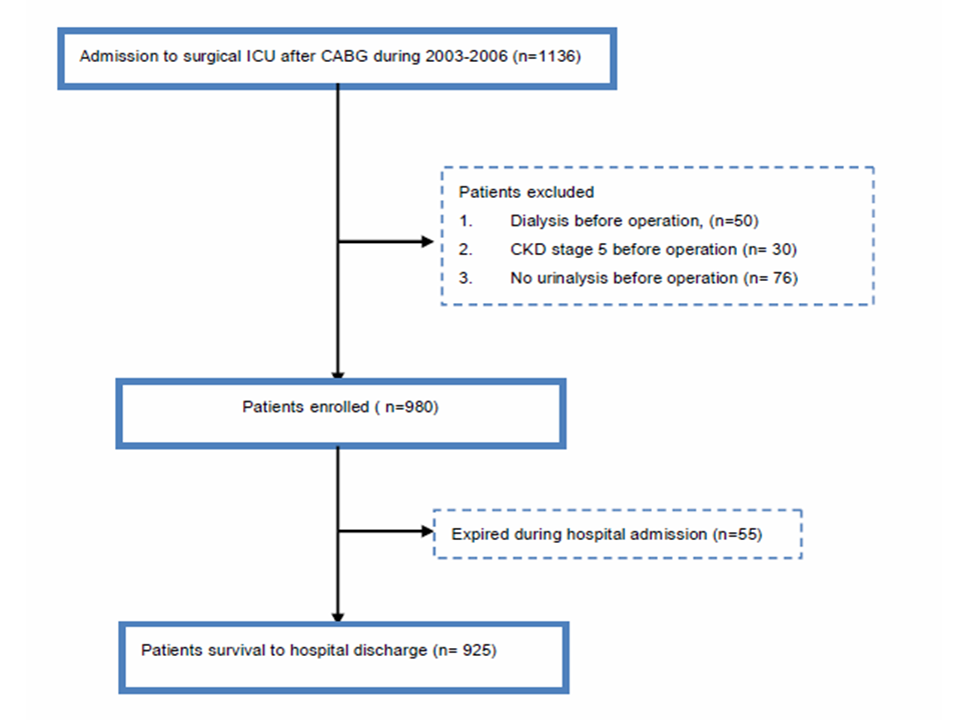

Supplement: Figure S1 — Flow diagram of the study population. ( AKI, acute kidney injury; CABG, coronary artery bypass grafting; CKD, chronic kidney disease; ESRD, end stage renal disease; ICU, intensive care unit). (TIF) [file pone.0027687.s001.tif]

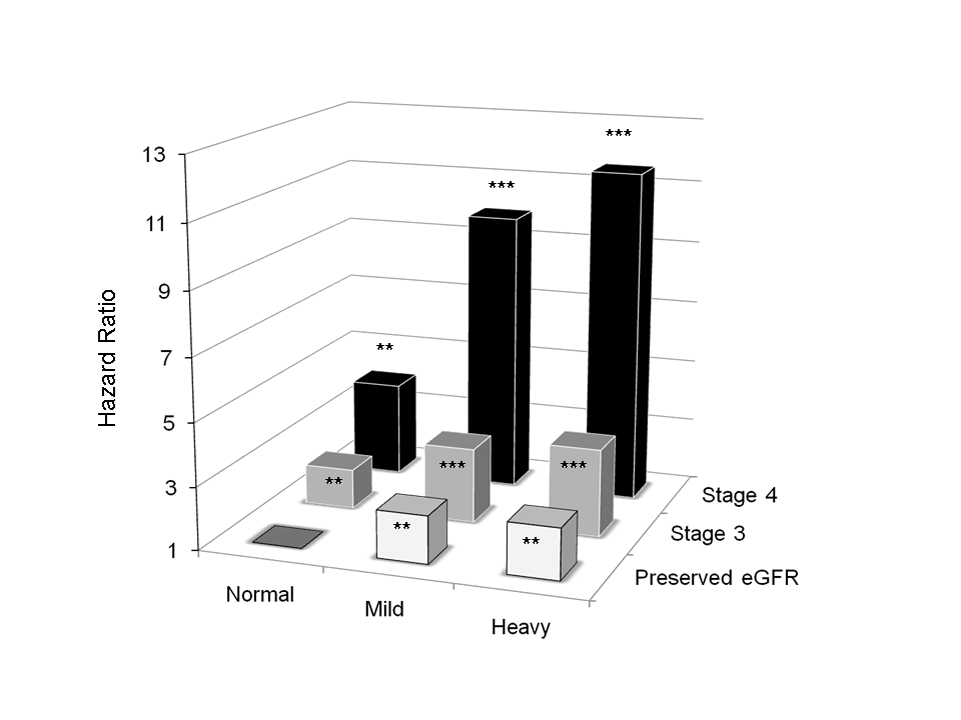

Supplement: Figure S2 — Hazard ratio (HRs) for the compo site outcome after hospital discharge (long- term end-stage renal disease or mortality) for urinary proteinuric categories across chronic kidney disease (CKD) categories. (adjusted for factors listed in Table 1. * p<0.05; ** p<0.01; and *** p<0.001 compared to patients with preserved eGFR and normal proteinuria). (TIF) [file pone.0027687.s002.tif]
