# Supplementary material for: Preoperative Proteinuria Is Associated with Long-Term Progression to Chronic Dialysis and Mortality after Coronary Artery Bypass Grafting Surgery
Source: PLoS One. 2012 Jan 20;7(1):e27687. doi: 10.1371/journal.pone.0027687 (PMC3262783; doi:10.1371/journal.pone.0027687)
Supplement: Table S1 — Percentage of patients in groups stratified by chronic kidney disease (CKD) stage and proteinuria. (DOCX) [file pone.0027687.s003.docx]

**Supplementary material**

Table S1. Percentage of patients in groups stratified by chronic kidney disease (CKD) stage and proteinuria

| **CKD Stages\ proteinuria**  **( % total patients)** | **Normal (n=530)** | **Mild proteinuria (n=276)** | **Heavy proteinuria (n=119)** |
| --- | --- | --- | --- |
| **Preserved eGFR ( 554)** | 373 (40.3%) | 147 (15.9%) | 34(3.7%) |
| **Stage 3 (319)** | 144 (15.6%) | 117 (12.6%) | 58(6.3%) |
| **Stage 4 (52)** | 13 (2.5%) | 12 (1.3%) | 27(2.9%) |

- Preserved eGFR: estimated glomerular filtration rates (eGFR) ≥ 60 ml/min/1.73m^2^; stage 3: 30 to 59.9 ml/min/1.73m^2^; stage 4: 15 to 29.9 ml/min/1.73m^2^
