# Supplementary material for: Preoperative Proteinuria Is Associated with Long-Term Progression to Chronic Dialysis and Mortality after Coronary Artery Bypass Grafting Surgery
Source: PLoS One. 2012 Jan 20;7(1):e27687. doi: 10.1371/journal.pone.0027687 (PMC3262783; doi:10.1371/journal.pone.0027687)
Supplement: Table S2 — Factors associated with long- term end stage renal disease (N = 925). (DOCX) [file pone.0027687.s004.docx]

Table S2. Factors associated with long- term end stage renal disease (N = 925)

| **Covariate** | **Hazard Ratio (95% CI)** | ***p* value** |
| --- | --- | --- |
| **Charlson score (/per)** | 1.30 (1.12– 1.51) | <0.001 |
| **Post CPR (yes)** | 5.18 (1.31– 20.41) | 0.019 |
| **Moderate/Severe MR** | 4.36 (1.94–9.80) | <0.001 |
| **Preoperative Proteinuria** |  |  |
| **No proteinuria** | 1 | - |
| **Mild proteinuria** | 2.83(1.01 – 7.99) | 0.049 |
| **Heavy proteinuria** | 27.17 (8.77– 84.15) | <0.001 |
| **CKD Stages** |  |  |
| **Preserved eGFR** | 1 |  |
| **Stage 3** | 10.71 (0.93 – 123.20) | 0.057 |
| **Stage 4** | 91.21 (31.05– 267.94) | <0.001 |
| **Joint probabilities across categories** | | |
| **Proteinuria*CKD stages** | 0.04 (0.01–0.175) | <0.001 |
| **R^2^= 0.183, df= 8, Goodness -of -fit assessment = 0.416** | | |

* Abbreviations: CI: confidence interval; CKD: chronic kidney disease; CPR, Cardiopulmonary resuscitation; MR, mitral valve regurgitation; TPN: total parenteral nutrition
