# Supplementary material for: Preoperative Proteinuria Is Associated with Long-Term Progression to Chronic Dialysis and Mortality after Coronary Artery Bypass Grafting Surgery
Source: PLoS One. 2012 Jan 20;7(1):e27687. doi: 10.1371/journal.pone.0027687 (PMC3262783; doi:10.1371/journal.pone.0027687)
Supplement: Table S3 — Factors associated with long- term all- cause mortality (N = 925). (DOCX) [file pone.0027687.s005.docx]

**Table S3. Factors associated with long- term all- cause mortality (N = 925)**

| **Covariate** | **Hazard Ratio (95% CI)** | ***p* value** |
| --- | --- | --- |
| **Age (years)** | 1.05 (1.03– 1.07) | <0.001 |
| **Proteinuria** |  |  |
| **No proteinuria** | 1 | - |
| **Mild proteinuria** | 1.88 (1.27– 2.80) | 0.002 |
| **Heavy proteinuria** | 2.28 (1.42– 3.66) | 0.001 |
| **CKD Stages** |  |  |
| **Preserved CKD stage** | 1 |  |
| **Stage 3** | 1.53 (1.20– 2.28) | 0.040 |
| **Stage 4** | 1.88 (1.03– 3.43) | 0.039 |
| **Hypertension ( yes)** | 0.49 (0.35– 0.70) | <0.001 |
| **Hemoglobin (g/dL)** | 0.86 (0.77– 0.96) | 0.010 |
| **Tracheostomy ( yes)** | 3.68 (2.02– 6.70) | <0.001 |
| **Elective Operation ( yes)** | 0.52 (0.35– 0.78) | <0.001 |
| **Post operative AKI ( yes)** | 1.66 (1.13– 2.44) | <0.001 |
| **Low LVEF** | 2.31 (1.58– 3.37) | <0.001 |
| **R^2^= 0.232, df= 11, Goodness -of -fit assessment = 0.267** | | |

✝✝ Abbreviations: AKI, acute kidney injury; CAD, coronary artery disease; CKD: chronic kidney disease; DM, diabetic mellitus; eGFR, estimated glomerular filtration rate; LVEF; left ventricular ejection fraction; RRT, renal replacement therapy.
