# Supplementary material for: Preoperative Proteinuria Is Associated with Long-Term Progression to Chronic Dialysis and Mortality after Coronary Artery Bypass Grafting Surgery
Source: PLoS One. 2012 Jan 20;7(1):e27687. doi: 10.1371/journal.pone.0027687 (PMC3262783; doi:10.1371/journal.pone.0027687)
Supplement: Table S4 — Factors associated with long- term composite outcome (N = 925). (DOCX) [file pone.0027687.s006.docx]

**Table S4. Factors associated with long- term composite outcome (N = 925)**

| **Covariate** | **Hazard Ratio (95% CI)** | ***p* value** |
| --- | --- | --- |
| **Age (years)** | 1.03 (1.01– 1.05) | 0.001 |
| **Proteinuria** |  |  |
| **No proteinuria** | 1 | - |
| **Mild proteinuria** | 2.57 (1.68 – 3.91) | <0.001 |
| **Heavy proteinuria** | 2.70 (1.69– 4.33) | <0.001 |
| **CKD Stages** |  |  |
| **Preserved CKD stage** | 1 |  |
| **Stage 3** | 2.24 (1.40– 3.57) | 0.001 |
| **Stage 4** | 3.52 (2.09– 5.94) | <0.001 |
| **Hypertension (yes)** | 0.57 (0.41– 0.80) | <0.001 |
| **IABP ( yes)** | 0.50 (0.28– 0.90) | 0.020 |
| **Elective operation ( yes)** | 0.55 (0.37– 0.80) | 0.002 |
| **Post operative AKI** | 1.91 (1.33– 2.76) | <0.001 |
| **Low LVEF** | 1.98 (1.40– 2.80) | <0.001 |
| **Tracheotomy (yes)** | 4.29 (2.24– 8.21) | <0.001 |
| **Hemoglobin (per g/dL)** | 0.87 (0.79– 0.96) | 0.006 |
| **Joint probabilities across categories** |  |  |
| **Proteinuria*CKD stages** | 0.52 (0.27– 0.99) | 0.045 |
| **R^2^= 0.277, df= 13, Goodness -of -fit assessment = 0.304** | | |

* Abbreviations: AKI, acute kidney injury; CI: confidence interval; CKD, chronic kidney disease; IABP: intra-aortic balloon pumping; LVEF: left ventricular ejection fraction; OR: odds ratio
